# Three Surgical Myths

**Published:** 1915-05

**Authors:** Thos. H. Hancock

**Affiliations:** Atlanta, Ga.


### "THREE SURGICAL MYTHS."

By Thos. H. Hancock, Atlanta, Ga.

This title would indicate something mysterious, but the contents of the paper are very plain. It has been written entirely from personal experience. The three surgical myths I wish to present are permanent spinal concussion, permanent displacement of the normal uterus by external violence and permanent sprain of the sacro-iliac synchondrosis.

Some years ago a surgeon published a book setting forth the sequelæ of injuries received in railway wrecks which were peculiar to themselves, but experience has not supported him.

It seems strange for one to set up a theory that because a person is injured in a railway wreck that his condition is different from one who is injured in a game of foot ball.

This theory was intended to apply only to those cases which were injured in wrecks where there was great fright and confusion, but it was at once applied to all railway accidents, and a peculiar circumstance was that it was attributed almost entirely to men, not because they were more easily frightened than women and not because they were not protected by corsets, but more particularly because they had no wombs to be misplaced.

Permanent spinal concussion, how mysterious, how awful to be injured in the spine!

The spine is twisted, wrenched and sprained we are told; the ligaments, muscles, sinews, tendons and nerves are torn, lacerated and permanently disorganized; the man suffers and will continue to suffer during the remainder of his life and he is disabled from earning a living by at least seventy-five per cent.

This is a frequent declaration in damage suits, and yet there may be no external evidence of injury, the symptoms may be entirely subjective.

This is no exaggeration of thousands of cases that have gone before juries of this country. Some of these plaintiffs have been carried into court on stretchers or in chairs and without having any objective signs.

Dermography, demonstrated by drawing a finger across the back or chest and producing thereby a red line, was offered at one time as a symptom of lesion of the cord, but experience soon showed that many people presented this condition who had never received any spinal injury and who were in perfect health. In fact, any one with a thin, delicate skin will show it. Nor does Westphal's sign, diminution or loss of the patella reflex show anything without other symptoms. A large number of perfectly healthy people have almost an absence of this reflex and also a large number have it exaggerated.

Anaesthesia or hyperaesthesia are easily simulated and are often of no value on this account.

They are without objective signs and yet in a permanent pitiable condition! We are intelligent men and have devoted out lives to the study of disorders and yet we can not tell the injured from the malingerer, we can not tell the honest from the dishonest. What a mockery to our profession, if we let this go unchallenged or unproved.

What has become of these hundreds of individuals that have suffered from this dreadful injury! What institutions are harboring them and what physicians are giving them attention?

We are unable to find one of them who has not made a speedy and perfect recovery as soon as the "green-back poultice has been applied. If the case has been thrown out of court, the same result has followed. A prominent neurologist asked me on one occasion to tell him what a green-back poultice was.

One of the local corporations has a record of a large number of these cases and the occupations that the men are now following. They have all recovered where there were no objective signs. A young man, who is now a chauffeur and stays around the Atlanta Terminal Station, had his back fractured in the lower part of the dorsal region several years ago. The condition was caused by a train running over him and he had no spinal concussion.

Some one has well said: "Facts are stubborn things."

It would not be possible in a paper of this length to go into the various traumatic neuroses, hysteria, sprains, etc. We are dealing now with surgical myths, one of which is spinal concussion without objective signs.

Another peculiar circumstance in reference to claims for damages on account of spinal concussion as previously mentioned is the fact that few of these claims are made for females who have displacements of the uterus instead. I know of only two and in both of these cases a verdict was found for the defendants.

None of the recent books mention cases of displacement of the uterus from external violence I believe, and it will be hard to get any proof that such can be the case. I recall a very amusing suit where a principal of one of the public schools, a maiden lady, had been injured and her attorneys had called two specialists, two of the leading gynecologists of the city. One of them swore that she had an antiflexion and the other that she had a retroflexion. The defense had only one doctor, who did not even make an examination of her uterus when he found that her own doctors would disagree, and he had only to tell the jury the difference between antiflexion and retroflexion. A mistrial resulted and the case has not been in court again.

Every one of us has drawn down a uterus for a curettage or an examination, and I venture to state that rarely has one taken the trouble to replace it, leaving it entirely to nature to go back. Why then should a normal uterus get a permanent displacement from an external force? This subject was discussed at Old Point Comfort, Va., several years ago in the presence of about a hundred surgeons from all parts of the Southern states, and not one of them could recall such a case. Could this be on account of the fact that they were all railway surgeons?

Several weeks ago the City of Atlanta paid a seventeen year old girl a thousand dollars on account of an accident in which it was claimed that her womb had been displaced, a retroflexion with adhesions to the intestine. The top of a manhole had turned as she stepped upon it and she had gone into it catching upon her elbows. She had been operated upon and a ventral fixation made. The city attorney was satisfied that the accident did not cause the condition, but he said this was the safest way out of the trouble.

Last year I saw a case of complete procidentia in a woman who claimed her condition was caused by stumbling over some loose bricks on a sidewalk. I could mention case after case in which the premises were as unreasonable.

It seemed though that both of these allegations, the spinal concussion and the displaced wombs, were getting too common, the juries were beginning to doubt them, and something new must be found under the sun, so an enterprising surgeon several years ago described a permanent sprain of the sacro-iliac joint and you would marvel at the number of cases that have sprung up since that time. It seems to have been contagious. Verily, the love of money is a root of evil.

There is no valid reason why this joint should have a permanent sprain more than any other joint. It is certainly one of the rarest sprains. I have seen a case where there was a complete separation of this joint and also motion between the pubic bones where there was no suit and where recovery was perfect.

When will doctors cease to disagree, when will we be able to mix soda and vinegar in the same water without commotion?

Man's unfairness to corporations has caused millions of dollars to be misappropriated.

Our President told us at our last meeting in his inaugural address that we ought to get together, that we ought to put our arms around and support each other, that envy, hatred malice and all uncharitableness should be things of the past. He spoke of the great advantage we would have by teaching and being taught. We, as brother physicians, should get together.

Our judges, from time to time, have selected physicians from our ranks who were unbiased, men who were learned and honest, so that they might know the truth in cases of dispute and I believe that the day is not far distant when this society will be called upon to settle these disputed points, when the plaintiff, the defendant and the juries will have confidence in the integrity of this body and will be willing for its decisions to be final.

Then will personal pride and personal gain be laid aside and we will be able to look at conditions with unbiased minds and undimmed sight. Let us be honest with our patients, let us be honest with ourselves.

We should get together!

